# Asymmetric competition over calling sites in two closely related treefrog species

**DOI:** 10.1038/srep32569

**Published:** 2016-09-07

**Authors:** Amaël Borzée, Jun Young Kim, Yikweon Jang

**Affiliations:** 1 Laboratory of Behavioral Ecology and Evolution, School of Biological Sciences, Seoul National University, 151-742, Republic of Korea; 2 Department of Life Sciences and Division of EcoScience, Ewha Womans University, Seoul, 120-750, Republic of Korea

## Abstract

Interspecific competition occurs when one species using a resource limits the use of that resource by another species. A dominance relationship between the species competing over a resource may result in asymmetric competition. Here, we tested the hypothesis that two sympatric treefrog species, the endangered *Hyla suweonensis* and the abundant *H. japonica*, compete with each other over calling sites. We observed the locations of calling individuals of the two treefrog species in rice paddies and tested whether removing one species affected the calling locations of the other species. Individuals of the two species were spatially isolated within rice paddies, with *H. japonica* at the edges and *H. suweonensis* in the interior. Male *H. suweonensis* moved towards the edges of rice paddies when male *H. japonica* were removed from the area, whereas male *H. japonica* hardly moved when male *H. suweonensis* were removed. The results of both studies are consistent with asymmetric interspecific competition, in which the calling locations of *H. suweonensis* are affected by the calling activity of *H. japonica*. In addition, *H. japonica* were found “sitting” on the substrate during call production, whereas *H. suweonensis* were “holding” onto vegetation. The difference in calling posture may represent an adaptive response to asymmetric interspecific competition.

When two or more species share a common resource, the consumption of that resource by one of the species creates a “resource depletion zone” for the other species (*sensu* Schluter^1^). Such competition may lead to interspecific niche partitioning^2^. Competition is asymmetrical when a dominant species limits the other species’ access to a resource. In amphibians, the salamander larvae *Ambystoma talpoideum* and *A. maculatum* compete with each other over food, and the latter species has a lower survival rate in the presence of the former^3^. Asymmetrical competition in the context of amphibian breeding ecology is well illustrated by green frogs (*Rana clamitans*) spawning closer to the edges of ponds when in the presence of bullfrogs (*R. catesbeiana*)^4^. In order to be subject to competition, two or more competing species must share a common resource, and resource use by one species should affect that of the other species, as this paper aims to exemplify in the framework of asymmetric competition in amphibian breeding assemblages.

The nature of interspecific competition is diverse in adult amphibians and often occurs over food, habitat, or acoustic space used for signalling^5^. Males of different species in a single location may defend their resources by using ritualised displays, such as calls^6^,^7^,^8^,^9^, thus avoiding direct physical contact^5^. The possible consequences of interspecific competition include temporal or spatial segregation between species, leading to niche partitioning^1^,^10^,^11^,^12^. A niche is defined as a localised microhabitat with biotic and abiotic variables that are different from those of the habitat as a whole. Niche segregation can be used as a tool to detect interspecific competition.

Two treefrog species are present in the Republic of Korea: *Hyla japonica*, widespread throughout North East Asia, and *H. suweonensis*, present entirely within the distribution of *H. japonica* on the western coastal plains of Korea. *Hyla suweonensis* is potentially synonymous to *H. immaculata*^13^, and it was recently suggested that its genus name be changed to *Dryophytes*^14^. The two species diverged between 3.2 and 7.1 million years ago^15^,^16^. Their advertisement calls are structurally similar, consisting of a train of single notes, but differ in call properties. The note repetition rate is slightly higher and the dominant frequency slightly lower in *H. japonica* than in *H. suweonensis*^17^,^18^. Distinguishing these two species based on morphology is difficult, although male *H. suweonensis* are more slender than male *H. japonica*^19^. Both treefrog species are primarily found in rice paddies, as natural wetlands adequate for breeding are extremely rare^20^. These two treefrog species are found together at the same time and location^21^.

Male treefrogs typically produce advertisement calls on the ground, branches, or tree trunks. In a lone semi-natural habitat for *H. suweonensis*, males of both species were calling at the edge of the wetland^20^. However, male *H. suweonensis* generally call in the middle of rice paddies and *H. japonica* males call at the edge of rice paddies^22^. The levee of rice paddies are typically composed of soils with grasses, which provide solid sites for calling treefrog males. During the breeding season, the middle of rice paddies is flooded and provides no solid ground for call production. Thus, it has been argued that the levee is an ideal location for producing advertisement calls, compared to other parts of a rice paddy. Furthermore, when female treefrogs move towards the rice paddy in order to mate and spawn, calling males located on a levee are likely to be the first to encounter females. Thus, male treefrogs prefer the levees of rice paddies when producing advertisement calls.

In the Republic of Korea, rice paddies are typically clustered together for ease of irrigation and management. A rice-paddy complex refers to a contiguous assemblage of paddies, which are divided by narrow banks, levees, or one-lane roads. Here, we used field observations and a removal experiment to investigate whether niche segregation occurs between *H. japonica* and *H. suweonensis* in rice paddies. Niche segregation for calling sites was assessed within rice paddies and within rice-paddy complexes, where similar rice paddies are typically grouped together. Niche segregation at calling sites may simply reflect differential use of time and space in a rice paddy by the two treefrog species. However, competition between the two species may affect the calling locations of one or both of the sympatric species. Thus, we hypothesised that the removal of one of the species would be correlated with an expansion in the range of calling sites used by the other. At the rice-paddy complex scale, we expected commonalities in the distribution of the two species in terms of landscape use, owing to similarities in their general ecological requirements.

## Results

### Observations within a rice paddy

**Figure 1:**
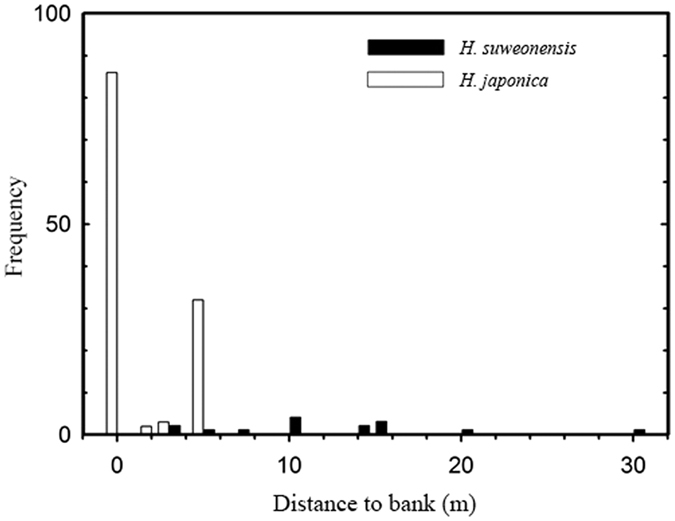
Distributions of distance to bank for *H. suweonensis* (solid, *n* = 15) and *H. japonica* (open, *n* = 123) in rice paddies. At night, when male treefrogs produced advertisement calls, they distributed themselves on the rice paddy banks or inside rice paddies. “Distance to bank” was the distance between the bank and the location of a calling treefrog. When a treefrog called on the bank, distance to bank was zero.

To understand niche segregation, we documented the locations of calling individuals of the two treefrog species through acoustic monitoring. When males of both *Hyla suweonensis* and *H. japonica* produced advertisement calls in the same rice paddy during the breeding season, they tended to be segregated from each other. At all locations, calling *Hyla japonica* (*n* = 123) were located on average 1.4 ± 2.0 m (mean ± SD) from the bank, while calling *H. suweonensis* (*n* = 15) were on average 12.1 ± 6.9 m from the bank (Fig. 1). The average difference between *H. japonica* and *H. suweonensis* calling sites within a rice paddy was 8.1 ± 3.9 m (min = 3; max = 14). The results of the Generalised Linear Mixed Model indicated that there was a significant difference between the two species regarding the distance to the bank, but not for individual, season, time of day, paddy size, or presence of road or ditch (Table 1). In a non-parametric analysis, *H. japonica* and *H. suweonensis* significantly differed in the location of calling sites within rice paddies (Mann-Whitney *U* test; *U* = 83.00; *n* = 138; *P* < 0.001).

The results of the statistical analysis of the distributions of the two species at the spatial scale of rice-paddy complexes showed that the two species were not significantly different in the likelihood of calling near a ditch (*χ*2 = 3.86, *df* = 2, *P* = 0.145) or a road (*χ*2 = 1.29, *df* = 1, *P* = 0.257). Thus, the preference for landscape features was analysed together for both species. Individuals of the two treefrog species (*n* = 138) tended to be present in rice paddies with natural ditches (52.9%) or without ditches (36.2%), and tended to avoid rice paddies bordered by concrete ditches (10.9%). Individuals of the two species were more likely to be found in rice paddies that were not adjacent to roads (74.6%), rather than in rice paddies adjacent to roads (25.4%).

### Observation within a rice-paddy complex

The purpose of this analysis was to assess the effect of landscape variables on the distribution of the two species. If niche segregation occurred at the level of a rice-paddy complex, only individuals of one treefrog species would be found in a given rice paddy. However, the frequencies of paddies with both or neither *Hylid* species present were consistently higher than expected in all four rice-paddy complexes, and the frequency of either species being present alone was consistently lower than expected (Table 2). The distribution of the two treefrog species differed significantly among the four rice-paddy complexes (Mantel-Haenszel common odds ratio: *ln*(estimate) = 3.197, standard error of *ln*(estimate) = 0.469, *P* < 0.001). Thus, we tested the hypothesis of niche segregation between the two treefrog species separately for each rice-paddy complex (Table 2). The presence of *H. suweonensis* was not independent of that of *H. japonica* in any of the four rice-paddy complexes (*G* test, likelihood ratio ≥ 8.630, *df* = 1, *P* ≤ 0.003). Therefore, frogs of both species were found to be using the same landscape elements at the rice-paddy complex scale. Thus, both treefrog species tend to prefer the same rice paddies and avoid others.

Next, we examined factors important for the presence of the two treefrog species within a rice-paddy complex. Because the number of rice paddies with *H. suweonensis* only was very small, these analyses were conducted for rice paddies with *H. japonica* only or for those with both species. The results of multinomial logistic regressions suggested that forest was a significant factor for the presence of *H. japonica*, and for that of both species. This is explained by treefrogs being more abundant in rice paddies close to forests in complexes 2 and 4 (Table 3). In these complexes, rice paddies were located closer to forests than to bushes. Paddies occupied by one or both species were on average 116.6 ± 86.1 m from forests and 139.6 ± 114.2 m from bushes in complex 2 and 61.6 ± 53.6 m from forests and 165.8 ± 127.1 m from bushes in complex 4. In complex 5, however, occupied paddies were, on average, further away from forests (distance to forest: 190.8 ± 159.7 m) than from bushes (distance to bushes: 70.4 ± 64.2 m), and distance to bushes was significantly associated with the presence of *H. japonica* and of both species. Distance to forests and distance to bushes differed significantly (*t* = 4.01, *df* = 516, *P* < 0.001). Roads tended to be significant for the locations of *H. japonica* and of both species in complexes 4 and 5, and treefrogs tended to call further away from roads in these complexes. Overall, calling location within a rice-paddy complex did not seem to follow a consistent pattern relative to landscape factors.

### Removal experiment

**Figure 2:**
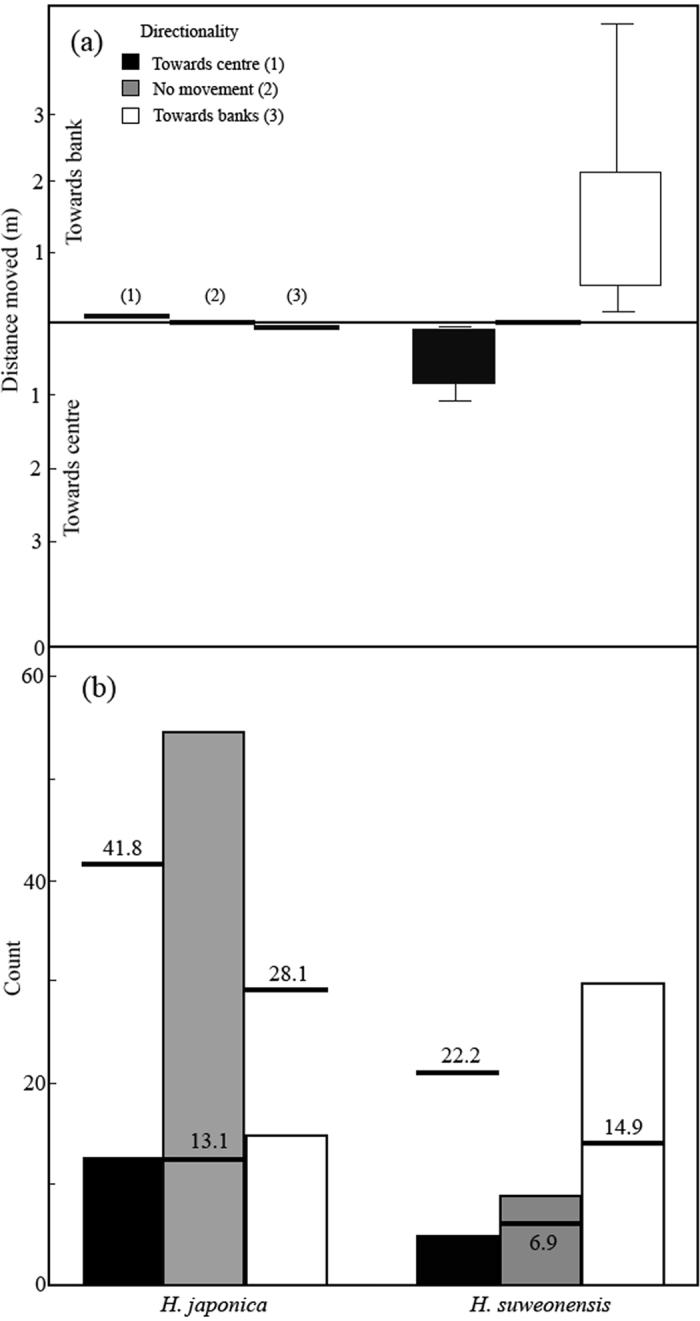
(**a**) Boxplots of the distance moved for the two treefrog species in the removal experiment. Calling locations of the focal species (either *H. japonica* or *H. suweonensis*) were noted in rice paddies before and after individuals of the non-focal species were removed. Direction of movement was measured as “towards the bank,” “no movement,” or “towards the centre of rice paddies”. (**b**) Observed counts (bars) of movement in the two treefrog species. Horizontal lines represent the expected counts based on the assumption that the two species did not differ in the direction of movement.

In each rice-paddy complex, two rice paddies with calling treefrogs of both species were randomly chosen. *H. japonica* was removed from one and *H. suweonensis* was removed from the other. We measured the distance to the closest bank for each individual, pre- and post-removal, to assess the impact of each species on the other one. When calling individuals of the opposite species were removed, focal individuals tended to move towards the position where the opposite species used to be. When *H. suweonensis* males were removed, 18.1% of calling *H. japonica* males moved towards the centre of rice paddies, while 15.7% moved towards the bank and 66.3% did not display any movement (Fig. 2b). When calling *H. japonica* males were removed, 11.4% of calling *H. suweonensis* males moved towards the centre of rice paddies, whereas 68.2% moved towards the bank and 20.5% did not display any movement. The directionality of the movement post-removal was statistically analysed through a likelihood ratio test. The two treefrog species differed significantly in the directionality of movement in the removal experiment (likelihood ratio test: χ^2^ = 36.71, *df* = 2, *P* < 0.001; Fig. 2a). The two treefrog species moved in the expected directions in the removal experiment, and the distance moved differed significantly between the two treefrog species (excluding individuals not displaying any displacement: *t* test; *t* = 8.54, *df* = 62, *P* < 0.001; including all individuals: *t* = −6.42, *df* = 125, *P* < 0.001). Calling *H. japonica* males barely moved in either direction in the removal experiment (0.35 ± 0.39 m, mean ± SD; range: 0.06–0.87 m; Fig. 2a). However, calling *H. suweonensis* males moved an average of 1.34 ± 1.24 m (mean ± SD, range: 0.08–4.76 m) towards the bank. The results of the regression analysis of movement before and after removal showed that only two factors, focal species and time after sunset, were significant (Table 4).

In summary, the calling locations of the two treefrog species were segregated within a rice paddy. Calling *H. japonica* were found on or close to banks, whereas male *H. suweonensis* called from the centre of rice paddies. Male *H. suweonensis* tended to be affected by the presence of *H. japonica*, but not the other way around.

### Calling posture

Binary logistic regression analysis was used to determine the factors affecting the calling posture during the production of advertisement calls. The factors analysed were species, snout-vent length (SVL), site, and season. Of 240 *H. japonica* individuals, 239 were “sitting” when producing advertisement calls, whereas 28 of 29 male *H. suweonensis* were “holding.” The results of the binary logistic regression showed that the only variable that explained significant variation in calling posture was species (Table 5), while season, site, and SVL were not significant. That is, the calling posture was significantly different between the two treefrog species. Anecdotally, male *H. suweonensis* produce advertisement calls while sitting on mud above water level in a rice paddy during the period between flooding and rice planting. However, we rarely observed male *H. japonica* holding onto plants while calling, even if they were sitting on the plants instead of the ground.

## Discussion

The results of our observational studies clearly demonstrate niche segregation between *Hyla japonica* and *H. suweonensis* over calling sites within individual rice paddies. The spatial separation between calling males of the two treefrog species within a rice paddy might be of competitive nature. The calling locations of *H. suweonensis* males were affected by the removal of calling *H. japonica*, but not the other way around. This result of the removal experiment supports interspecific competition over calling sites in the two treefrog species. Furthermore, the alternative hypothesis, that the differential use of non-calling site resources in rice paddies between the two treefrog species may result in niche segregation in calling sites is unlikely, as this hypothesis would predict no displacement of calling sites in the removal experiment. Accordingly, *H. suweonensis* males may predate on mayflies that occur mostly inside the rice paddies, whereas *H. japonica* males feed on non-flying arthropods at the banks of rice paddies. If this hypothesis is true, male movements of either species in and around rice paddies should be correlated with prey density, not with the absence of advertisement calls made by interspecific competitors.

It is unclear what fitness disadvantage may occur when calling male *H. suweonensis* approach the bank of a rice paddy where male *H. japonica* produce advertisement calls. Male *H. japonica* may be physically aggressive towards the calling *H. suweonensis* males. Whether the treefrog species directly compete with each other remains unclear. However, contact between individuals of these two species is rarely observed. Alternatively, the advertisement calls of *H. japonica* may interfere with those of *H. suweonensis* in close proximity. Although the fitness consequences of competition have not yet been determined, niche segregation and adjustment of calling sites in the absence of competitors are consistent with the predictions of interspecific competition between the two species.

Competition over calling sites between calling males of the two treefrog species was asymmetric. Male *H. japonica* are 12.9% larger than *H. suweonensis*^18^, and *H. japonica* is more abundant than *H. suweonensis* throughout the known range of *H. suweonensis*^21^. The physical superiority and abundance of *H. japonica* may provide a competitive advantage over the sympatric species, allowing *H. japonica* to dominate the banks of rice paddies for call production. The effectiveness of a male’s advertisement calls in attracting a female may be severely reduced by the loud background noise produced by a large group of *H. japonica* males. This may cause *H. suweonensis* to move away, towards a less ideal habitat, and consequently lead to reduced mating opportunities or interspecific mating. More studies are needed to strengthen the hypothesis of asymmetric competition between the two treefrog species over calling sites, including playback experiments in which simulated conspecific calls affect the calling locations of the two treefrog species.

The spatial arrangement of calling sites in a rice paddy resulted in *H. japonica* males calling at the edges of rice paddies, along a line parallel to the water line, because of territorial boundaries. This creates a “barricade” that females of the central species, i.e. *H. suweonensis*, have to cross before reaching conspecific males. This arrangement may decrease the number of females from the central species reaching the breeding area without interference from males of the edge species. This spatial arrangement is also seen for *H. gratiosa* and *H. cinerea*, two North American *Hylids*, where males of the former species call from the pond proper, and males of the latter species call from outside the water^23^.

Furthermore, because of the possibility of hybridisation^24^ and erroneous mate selection by females^25^, the spatial arrangement presents a challenge to *H. suweonensis* species integrity. The predominance of the *H. japonica* population may facilitate directional introgression during hybridisation^26^. In this case, if hybridisation occurs in the wild, male *H. japonica* mating with female *H. suweonensis* may predominate over the reciprocal combination. Such a directional bias may lead to backcrossing in later generations, thus leading to cytonuclear disequilibrium and creating introgression patterns comparable to those found in *Bufo viridis*^27^.

We did not detect any evidence of niche segregation with respect to time of day or season. Males of both species typically produce advertisement calls at the same time. Anecdotal observations (personal observations by A. Borzée and Y. Jang) suggest that *H. suweonensis* advertisement calls are almost always accompanied by *H. japonica* advertisement calls in the same rice paddy. Because this study was limited to a maximum of 32 consecutive days, there is a possibility of niche segregation in breeding seasons, outside of the study period. However, a more extensive, nine-week study showed that the calling seasons of both treefrog species completely overlap^21^. Thus, temporal separation is unlikely to be the mechanism isolating *H. japonica* and *H. suweonensis* populations.

Segregation of calling locations within a rice paddy may directly affect body posture during the production of advertisement calls. Male *H. suweonensis* call from the centre of rice paddies, where they cannot sit on a hard substrate and must hold onto rice seedlings. Conversely, male *H. japonica* mainly call from rice paddy banks, where they can sit on a substrate during call production. In the only semi-natural habitat known for *H. suweonensis*^20^, which is characterised by a network of marshes, grasses, and trees, male *H. suweonensis* assume a sitting posture during the production of advertisement calls^28^. The holding posture is exhibited in rice paddies, where *H. suweonensis* are forced into the centre of the water body because of competition from *H. japonica*. We believe that the sitting posture is preferred for *H. suweonensis* in the heterogeneous semi-natural habitat where *H. suweonensis* can avoid direct contact with *H. japonica*. If interspecific competition is driving the arrangement of calling locations in the two treefrog species in rice paddies, holding may be an adaptation for calling at the centre of rice paddies in *H. suweonensis*.

This study suffers from a lack of intraspecific controls in the removal experiment, a low *H. suweonensis* sample size, and a limited number of sampling areas. The effect of removal of intraspecific calling males should be compared to the effect of removal of interspecific calling males in a future removal experiment. Observational and removal studies should be replicated over areas that represent the whole range of *H. suweonensis*. Another potential problem of this study was that the spatial correlations of rice paddies within a rice-paddy complex may bias the effects of landscape features on the positioning of the two treefrog species.

Neither treefrog species was randomly distributed with respect to landscape features within a rice paddy. Within rice-paddy complexes, rice paddies with only *H. japonica* and with both species generally had similar landscape features. These findings suggest similar ecological requirements for both treefrog species in a rice-paddy complex. The only landscape features correlated with the presence of the two species were related to vegetation. Forest and bushes are hypothetical hibernation sites^29^, and breeding sites need to be within an average of 2000 m of these features^30^,^31^ for the annual migration between hibernating and breeding sites. The distance from rice paddies to the closest vegetation, defined as a combination of the forest and bush variables, was similar for both species. This result can be explained by a common need for shelter, resting places, and predator avoidance^32^.

The finding that both treefrog species tended to prefer the sides of rice paddies with no ditch or a natural ditch to those with a concrete ditch may be important for conservation of anuran species, including the endangered *H. suweonensis*. A tracking experiment showed that both treefrog species spent their days on rice paddy banks resting, hiding, and feeding^28^. Unlike natural ditches, concrete ditches typically remain dry, which limits grass growth. This finding differs from that of a study in Japan^33^, in which ditches did not have an effect on the temporal or spatial distribution of anuran species, including *H. japonica*. However, this study examined the presence of treefrogs in rice paddies, without examining which side of the rice paddies attracted the treefrogs. Typically, only one side of a rice paddy is bordered by a concrete ditch, so frogs can remain close to the sides of rice paddies without concrete ditches.

The information obtained in this study is critical for maintaining species diversity and conserving endangered *H. suweonensis*. Traditionally, differences in male advertisement calls and female selectivity are regarded as the driving forces for both speciation and maintenance of species diversity in anurans. However, asymmetric competition can influence population dynamics and community structure by affecting the population size of rare or endangered species^34^. In rice paddies, *H. suweonensis* occurs with other frog species, including *H. japonica*, the black-spotted pond frog *Pelophylax nigromaculatus*, and the Seoul golden frog *Pelophylax chosenicus*. Several frog species may compete with each other for calling locations, and dynamic interactions among species may determine breeding locations in a multi-species anuran community.

## Materials and Methods

### Study area and species

**Figure 3:**
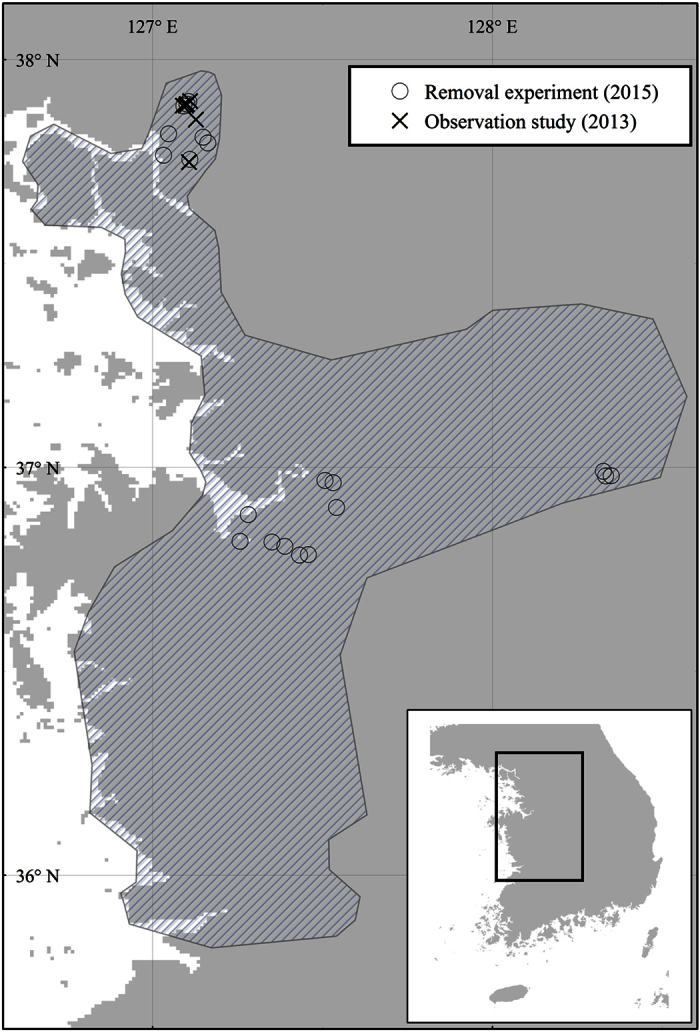
Map showing the range of *Hyla suweonensis* and the sampling sites used in this study. The species range (dashed area) is limited in the east by the elevation of the Taebaek mountain range and to the west by the Yellow Sea. *H. japonica* is present on all landmasses visible on the map. This map was generated with ArcMap 9.3 (Environmental Systems Resource Institute, Redlands, California, USA; http://www.esri.com/).

This research consisted of observational studies and a removal experiment. All experimental removals were approved and carried out in accordance with the guidelines of the Ministry of Environment of the Republic of Korea (permit numbers 2015–03, 2015–05, 2015–6, and 2015–28). The observational studies were conducted in the city of Paju, Gyeonggi province, and the removal experiment was conducted throughout the range of *Hyla suweonensis* in the Republic of Korea (Fig. 3). Anuran species at the study sites generally included *Pelophylax nigromaculatus*, *P. chosenicus*, *H. japonica*, and *H. suweonensis*. Adult *Rana coreana*, which are early spring breeders, were present, but they did not produce advertisement calls at our study sites during the study period. *H. japonica* spawns in shallow water between late April and early July, while *H. suweonensis* breeds from May to late June. Therefore, observational studies and removal experiments were conducted between 17 June and 1 July 2013 and between 19 and 28 June 2015, respectively. Both observational and experimental studies took place between 19:00 and 02:30 the next day, matching the peak calling activity of *H. japonica*^35^. The rice-paddy complexes we investigated were separated from each other either by more than 2000 m or by non-crossable landscape elements^36^,^37^ and were therefore considered independent for statistical analyses^21^.

Before the observational studies and removal experiments, we randomly selected rice paddies in rice-paddy complexes. To verify the presence of calling males of both species, we spent 5 min assessing the calling activity in each rice paddy prior to the experiment. If no calling activity was registered, the protocol was repeated until advertisement calls were detected from either Hylid species. No form of aggression between the two species has been published, and the only antagonistic relationship is assumed to be through advertisement calls^17^,^18^,^38^. All sampling was completed in rice paddies where treefrogs did not use floating plants or muddy patches above water level as substrates. During our study, the water depth was 20 cm on average, and the seedlings varied from 20 to 50 cm high. Patches above water level, later in the season, may affect the position of *H. japonica* within rice paddies. The location of females during the breeding season is not known, and was inferred from that of other Hylid species.

### Calling location within a rice paddy: observational study

To determine the niche segregation for calling location within a rice paddy, we conducted acoustic monitoring of the two treefrog species in 16 rice paddies that were randomly sampled from 6 rice-paddy complexes (Table 6a and Table 7). Each paddy in a complex was given a unique number, and rice paddies were randomly chosen for acoustic monitoring (Brandao Apps, 2010). The minimum distance between adjacent rice paddies was 85 m. Adjacent rice paddies less than 100 m apart were not sampled on the same night (Table 7).

Once a rice paddy was selected, LED markers (SY-MN-02; Xiamen Shangyi Technologies Co. Ltd.; Xiamen Fujian, China) were silently deployed on two adjacent banks of the rice paddy every three or five meters, with a maximum of 20 LED markers per rice paddy to prevent disturbance. The intensity of each LED marker was set at 3.09 (±0.53) lux at 1 m. The effect of the LEDs on chorus dynamics was investigated before and after recording, and the number of individuals calling did not differ significantly depending on the presence of the markers (two-sided *t* test; *t* = 0.194, *df* = 15, *P* = 0.849). Following the setting of these markers, a Sony PCM D-50 recorder fitted with a directional microphone (Sennheiser ME62 + K6 powering module, frequency response: 20–20000 Hz ± 2.5 dB; Wedermark, Germany) covered with a wind screen (MZW20-1 Blimp Windscreen; Sennhwiser; Wedermark, Germany) was used to record along two continuous banks, by pointing towards the opposite bank. The microphone position relative to the markers was recorded to provide evidence for calling activity at a given location in the rice paddy. In addition, two people noted the locations at which frogs were calling on previously prepared maps of the rice paddy. Each person was assigned a separate side of the rice paddy, and the distance from the bank to the location of the calling individual was assessed using the LED markers. The minimum listening period was five min, with minor variations owing to the time needed to walk around the rice paddy. The results obtained from the two maps and the recordings were compared, and only matching data were used for further analysis.

After the observations, we noted the physical characteristics of the rice paddies, including their surfaces. The presence of the following characteristics was also noted for each rice paddy: vegetation, one-lane roads, and ditches. Vegetation had to be at least 30 cm thick and high to be recorded. A one-lane road was defined as a paved road used for agricultural vehicles, with infrequent automobile use. Ditches were used for irrigation, and a rice paddy was in contact with a ditch when surrounded by other rice paddies. Concrete ditches had a rectangular cross section and were free of any vegetation, whereas natural ditches were vegetated, regardless of the steepness of their banks.

### Calling location within a rice-paddy complex: observational study

To determine niche segregation between the two treefrog species within a rice-paddy complex, we sampled four rice-paddy complexes in Paju (Table 6). The number of rice paddies in a complex ranged from 59 to 284, with areas between 0.21 and 1.41 km^2^. Each complex was investigated for 60 min at a randomly selected time between 16:00 and 05:00 the next day, for a minimum of eight times between 30 May and 1 July 2013. Calling activity was assessed independently for both species for each rice paddy in a complex. Each time a call was heard, the rice paddy from which the call originated was noted on the map. If several individuals were recorded calling throughout the study period, only one mark was noted on the map.

We measured the following physical characteristics of all rice paddies in the complexes: distance to bush, distance to forest, and distance to road. Distance to bush was defined as the distance from the centre of each rice paddy to the closest vegetation between 30 and 60 cm high and up to 50 cm wide and long. Smaller bushes were excluded from the analysis as they were not constant throughout the breeding season because of agricultural practices. Distance to forest was defined as the distance from the centre of each rice paddy to the closest vegetation with a minimum height of 60 cm and a minimum width of 50 cm, here defined as forest. Due to the remoteness of forest for complex 1, which was farther than the average dispersal distance for treefrogs^30^, we arbitrarily assigned the distance to forest as 5000 m for complex 1. Distance to road was the distance from the centre of each rice paddy to the closest one-lane concrete or dirt road crossing the rice-paddy complex.

### Removal experiment

The removal experiment was conducted in 2015 because permits are only available every second year at any given site (Table 6). We used 20 rice-paddy complexes for this study. The sequence in which the two rice paddies were used was randomized. Distance to bank was defined as the distance between the frog and the water line of the closest bank. Distance to bank for all frogs of the focal species was measured with a range finder (SD 60; Sincon; Taichung, China), at 1 cm resolution. If a frog was less than 50 cm from the bank, a 5 cm resolution was used to prevent disturbance. Distance to bank was positive or negative if the individual was inside or outside, respectively, of the flooded area of the rice paddy. We then proceeded to temporarily remove all frogs of the non-focal species (maximum *n* = 11 for *H. japonica* and *n* = 4 for *H. suweonensis*). Once non-focal males were removed, advertisement calls were usually produced within 25–30 min at the location of removal by previously silent males or by males that moved from nearby paddies. Thus, a 20-min interval was selected before measuring the locations of the focal species following the same protocol. All frogs removed were released at the place of capture after the experiment.

### Calling posture

The body posture exhibited by calling frogs was investigated in 273 males. Because of the presence of satellite males, only calling males were included in the analysis. Eighteen sites were observed in six rice-paddy complexes between 5 May and 14 July 2013 (Table 6). Two types of body position were recorded. The first one, herein defined as “holding,” occurred when a frog had the digits of its forelegs curled around a bearing support (rice seedling) and its hind legs extended. In this position, frogs were usually not parallel to the water. “Sitting” was defined as a frog not using the digits of its forelegs to hold onto a substrate and having its hind legs in a resting position. Thus, when frogs were “sitting,” their bodies were always parallel to the substrate.

### Statistical analyses

To assess whether the calling sites of the two treefrog species were segregated within a rice paddy, we employed a Generalized Linear Mixed Model (GLMM) on the observation data from rice paddies, with distance to bank as a response variable. The predictor variables included season, time of day, species, individual, paddy size, road, and ditch. “Species” was either *H. japonica* or *H. suweonensis*. “Individual” referred to each individual frog, each one of which was assigned a random number, creating a nominal variable. “Paddy size,” “season,” and “time of day” were continuous variables. “Season” was the number of days since 16 April 2013, when advertisement calls for both species were heard for the first time that year. “Time of day” was the time after sunset at the closest weather post of the National Weather Service Stations of Korea on the day of monitoring. “Paddy size” was the surface area of a rice paddy, and “paddy width” was the width at which the distance-to-bank measurements were taken. “Road” denoted the absence (0) or presence (1) of a one-lane road, and “ditch” was divided into no ditch (0), natural ditch (1), or concrete ditch (2). Because distance to bank was not normally distributed (Kolmogorov-Smirnov test; *D* = 83.00, *df* = 138, *P* < 0.001), we additionally used the non-parametric Mann-Whitney *U* test to see whether *H. japonica* and *H. suweonensis* differed in their calling site locations. None of the variables were correlated with each other (*r* ≤ 0.16, *P* ≥ 0.059).

A binomial test was used to test the effects of road and vegetation on treefrog locations within a rice paddy. A null hypothesis of equal probabilities for the two categories was assumed for each binomial test. A Chi-square test was used to understand the effect of ditch, with an assumption of equal probabilities among the three categories.

To test the hypothesis of niche segregation at the level of a rice-paddy complex, we tabulated a 2 × 2 contingency table for each complex. Each rice paddy in a complex had one of four outcomes: both species present, both species absent, only *H. japonica* calling, and only *H. suweonensis* calling. We employed a *G* test of independence to determine niche segregation between the treefrog species in terms of calling location within a rice-paddy complex. The expected frequencies were calculated in a contingency table, assuming that calling males of the two treefrog species were independent of each other. To determine whether to pool data from the four complexes, we estimated the Mantel-Haenszel common odds ratio. We conducted multinomial logistic regression to determine the factors important for the presence of the two treefrog species. The response variable of the multinomial logistic regression was species presence (0: none; 1: *H. japonica*; 2: *H. suweonensis*; 3: both), and the predictor variables were distance to bush, distance to forest, and distance to road. Because the sample size for *H. suweonensis* was small, multinomial logistic regressions were conducted for *H. japonica* alone and for both species, but not for *H. suweonensis* alone.

In the removal experiment, an individual treefrog was assigned one of three categories for movement: “no movement,” “movement towards the centre of the rice paddy,” or “movement away from the centre of the rice paddy.” We counted the number of individuals in the three categories for each *Hylid* species. We employed a likelihood ratio test of independence to test whether the two treefrog species differed in the directionality of movement in the removal experiment. The expected frequencies of movement were based on the assumption that the two treefrog species did not differ in the directionality of movement. We then tested the difference in the distance moved pre- and post-removal of the non-focal species through ordinary least square regression analysis. The response variable was distance moved, and the predictor variables were focal species, site, frog ID, season, time after sunset, rice paddy width, and number of individuals removed. The reference day for the variable season was 5 May 2015. “Distance moved” was defined as the distance travelled by an individual before and after removal, and could be positive or negative depending on the position of the frog. “Number of individuals removed” was the number of individuals temporarily removed from the rice paddy during the experiment.

The factors included in the binary logistic regression analysis, conducted in order to determine the factors affecting calling posture during the production of advertisement calls, were: species, snout-vent length (SVL, measured with digital callipers 317–249; Mitutoyo Corp.; Kawasaki, Japan), site, and season. All statistical analyses were computed using SPSS v21.0 (SPSS, Inc., Chicago, IL, USA).

## Additional Information

**How to cite this article**: Borzée, A. *et al*. Asymmetric competition over calling sites in two closely related treefrog species. *Sci. Rep*. **6**, 32569; doi: 10.1038/srep32569 (2016).

## Figures and Tables

**Table 1:** Results of the Generalised Linear Mixed Model for niche segregation of calling location within rice paddies (*Hyla japonica, n* = 123; *H. suweonensis, n* = 15).

	df1	df2	F	P
Species	1	9	7.86	0.021
Individual	121	9	0.31	0.998
Season	1	9	0.05	0.834
Time of day	1	9	0.01	0.996
Paddy size	1	9	0.01	1.000
Road	1	9	0.09	0.774
Ditch	2	9	1.18	0.349

The response variable was distance to bank, and predictor variables were species, individual, season, time of day, paddy size, road, and ditch. Species was a nominal variable, and individual was a repeated measure.

**Table 2:** Distributions of the two treefrog species within the four rice-paddy complexes.

		*H. suweonensis*		
		absence	presence	sum
*H. japonica*	Complex 1 (Likelihood ratio = 13.52, *df* = 1, *P* < 0.001)			
	absence	37 (32.7)	24 (28.3)	61
	presence	0 (4.3)	8 (3.7)	8
	sum	37	32	69
	Complex 2 (Likelihood ratio = 21.25, *df* = 1, *P* < 0.001)			
	absence	121 (114.6)	59 (65.4)	180
	presence	0 (6.4)	10 (3.6)	10
	sum	121	69	190
	Complex 4 (Likelihood ratio = 8.63, *df* = 1, *P* = 0.003)			
	absence	20 (15.1)	26 (30.9)	46
	presence	2 (6.9)	19 (14.1)	21
	sum	22	45	67
	Complex 5 (Likelihood ratio = 117.28, *df* = 1, *P* < 0.001)			
	absence	140 (115.3)	89 (113.7)	229
	presence	3 (27.7)	52 (27.3)	55
	sum	143	141	284

The numbers in cells are the number of rice paddies with treefrogs in a rice-paddy complex, and the numbers in parentheses are the expected values.

**Table 3:** The result of multinomial logistic regression for factors important for the presence of treefrog species within rice-paddy complexes.

Factor	*H. japonica*			both species		
	*B* ± SE	Wald	*df*, *P*	*B* ± SE	Wald	*df*, *P*
Complex 1						
Bush	−0.14 ± 0.005	7.588	1, **0.006**	−0.027 ± 0.012	5.392	1, **0.020**
Road	0.027 ± 0.024	1.232	1, 0.267	0.013 ± 0.031	0.186	1. 0.666
Complex 2						
Bush	0.001 ± 0.002	0.069	1, 0.793	0.005 ± 0.003	2.305	1, 0.129
Road	0.019 ± 0.010	3.709	1, 0.054	0.007 ± 0.017	0.168	1, 0.682
Forest	−0.012 ± 0.002	23.824	1, **<0.001**	−0.010 ± 0.005	3.747	1, 0.053
Complex 4						
Bush	−0.011 ± 0.006	3.360	1, 0.067	−0.022 ± 0.007	10.346	1, **0.001**
Road	0.062 ± 0.027	5.159	1, **0.023**	0.055 ± 0.029	3.752	1, 0.053
Forest	−0.029 ± 0.011	7.571	1, **0.006**	−0.032 ± 0.011	8.965	1, **0.003**
Complex 5						
Bush	0.005 ± 0.003	2.978	1, 0.084	0.000 ± 0.004	0.002	1, 0.961
Road	0.043 ± 0.010	18.549	1, <**0.001**	0.023 ± 0.011	4.242	1, **0.039**
Forest	0.001 ± 0.001	1.110	1, 0.292	0.000 ± 0.001	0.017	1, 0.897

There are four possible combinations for the occurrences of the two treefrog species: *H. suweonensis* only, *H. japonica* only, both species, and neither *Hylid* species. Because the sample sizes for *H. suweonensis*-only populations were small, multinomial logistic regressions were conducted for *H. japonica* only and for both species. “Forest” could not be calculated for complex 1. Significant values are in bold.

**Table 4:** Linear regression analysis for the distance moved before and after removal of the non-focal species.

	Beta	Std. Error	*t*	*P*
Focal species	0.45	0.18	4.25	<0.001
Site	0.39	0.12	0.46	0.648
Frog ID	0.08	0.02	0.08	0.938
Season	−0.40	0.10	−1.15	0.250
Time after sunset	−0.18	0.00	−2.18	0.031
Rice paddy width	0.00	0.00	0.01	0.993
# of individuals removed	0.08	0.00	0.84	0.400

Predictor variables were focal species, site, frog ID, season, time after sunset, rice paddy width, and number of frogs removed.

**Table 5:** Binary logistic regression used to determine factors associated with the calling posture (*n* = 273).

Factor	*B*	Wald	*df*	*P*
Site	0.51	1.01	1	0.316
Season	−0.03	0.17	1	0.682
SVL	0.11	0.3	1	0.581
Species	−8.35	25.51	1	<0.001

The response variable was calling posture, and the predictor variables were site, season, snout-vent length (SVL), and species. There were two calling postures that the treefrogs adopted while producing advertisement calls, sitting and holding.

**Table 6:** Descriptions of rice paddies where the observation (a) and removal (b) experiments were conducted.

(a) Observation experiment								
Complex			Surface (km2)	# of paddies	# of paddies with			
	Coordinates (N & E in°)				*Hj* only	*Hs* only	both species	no Hyla
1	37.898	126.758	1.41	69	24	0	8	37
2	37.890	126.750	0.21	190	59	0	10	121
3	37.887	126.749	0.05	11	—	—	—	—
4	37.887	126.741	0.29	67	26	2	19	20
5	37.852	126.773	1.17	284	89	3	52	140
6	37.748	126.756	1.03	134	—	—	—	—
**(b) Removal experiment**								
**Sites**	**Coordinates (N & E in°)**		**#*****Hj*** **removed**	**# focal species**	**Paddy width (m)**	**#** ***Hs*** **removed**	**# focal species**	**Paddy width (m)**
1	37.754	126.758	2	2	21.45	3	2	34.00
2	37.795	126.802	12	2	28.90	1	9	34.05
3	37.809	126.790	84	3	27.77	1	4	35.38
4	37.817	126.706	3	3	27.30	1	5	37.42
5	37.897	126.755	7	1	57.43	2	11	44.84
6	37.890	126.751	2	1	8.36	1	4	18.58
7	37.885	126.743	9	2	9.90	1	2	77.14
8	37.765	126.693	3	1	53.11	2	4	42.47
9	36.884	126.901	33	4	40.90	2	1	45.70
10	36.818	126.881	18	2	29.29	3	2	44.76
11	36.962	127.109	4	2	19.02	2	4	54.07
12	36.967	127.089	74	3	30.20	1	2	57.83
13	36.901	127.118	17	5	32.67	3	9	27.32
14	36.990	127.772	4	2	50.93	1	5	54.28
15	36.978	127.777	2	1	26.70	1	2	31.96
16	36.979	127.791	4	1	107.97	1	1	39.93
17	36.786	127.048	11	2	52.41	4	8	97.79
18	36.784	127.027	7	2	72.93	1	3	18.53
19	36.806	126.991	7	4	111.61	1	1	28.83
20	36.816	126.959	1	2	36.65	2	4	40.53

*Hj* stands for *Hyla japonica*, and *Hs* stands for *H. suweonensis*. In the observation experiment, which was conducted in 2013, six rice-paddy complexes were used. All six were located in the city of Paju, province of Gyeonggi, Republic of Korea. Calling locations were sampled in 16 rice paddies from 6 complexes. Complexes 1, 2, 4, and 5 were sampled for calling location within rice-paddy complexes. “# Of paddies” denotes the number of rice paddies per complex with the presence of each or no *Hylid* species. The removal experiment, which was conducted in 2015, included 20 rice-paddy complexes. Two rice paddies at each site were randomly selected, one with *H. japonica* as the focal species and one with *H. suweonensis* as the focal species. Individuals of the non-focal species were temporarily removed from the rice paddy.

**Table 7:** Description of the 16 rice paddies surveyed for niche segregation of calling location within rice paddies.

Complex	Date	Time of day	Area (m^2^)	# of *H. japonica*	# of *H. suweonensis*
1	June 21	22:30	2718	7	1
1	June 21	22:00	4026	4	1
1	June 27	02:30	3848	8	0
1	June 27	22:30	6600	22	0
2	June 20	23:30	1249	0	2
3	June 21	00:00	2697	5	1
3	June 29	23:30	1040	10	0
4	June 17	20:00	3599	4	1
4	June 24	22:00	1350	1	0
4	June 24	22:45	5988	9	2
4	June 24	00:00	2086	4	0
4	June 25	00:30	1120	9	0
4	June 25	01:00	3442	13	0
5	June 28	22:45	3693	10	2
6	July 1	22:00	3055	0	2
6	July 1	23:30	4448	17	3

The 16 rice paddies represent all six complexes in the city of Paju. All surveys were conducted in 2013.
